# ETS1 and SP1 drive *DHX15* expression in acute lymphoblastic leukaemia

**DOI:** 10.1111/jcmm.13525

**Published:** 2018-03-07

**Authors:** Xiang‐Lei Chen, Yuan‐Hua Cai, Qiao Liu, Li‐Li Pan, Shui‐Ling Shi, Xiao‐Li Liu, Yuan Chen, Jing‐Gang Li, Jing Wang, Yang Li, Xiao‐Fan Li, Shao‐Yuan Wang

**Affiliations:** ^1^ Department of Hematology Fujian Institute of Hematology Fujian Provincial Key Laboratory on Hematology Fujian Medical University Union Hospital Fuzhou China; ^2^ Union Clinical Medical Colleges Fujian Medical University Fuzhou China

**Keywords:** DHX15, ETS1, promoter, SP1

## Abstract

*DHX15* plays a role in leukaemogenesis and leukaemia relapse. However, the mechanism underlying the transcriptional regulation of *DHX15* in ALL has not been elucidated. Our present study aimed to explore the functional promoter region of *DHX15* and to investigate the transcription factors controlling the transcription of this gene. A luciferase assay performed with several truncated constructs identified a 501‐bp region as the core promoter region of *DHX15*. Site‐directed mutagenesis, electrophoretic mobility shift and chromatin immunoprecipitation assays showed that ETS1 and SP1 occupied the *DHX15* promoter. Furthermore, knockdown of ETS1 and SP1 resulted in suppression of *DHX15*, whereas the overexpression of these genes led to up‐regulation of *DHX15*. Interestingly, in samples obtained from patients with ALL at diagnosis, both *ETS1* and *SP1* correlated positively with *DHX15* expression. Additionally, differences in methylation of the *DHX15* core promoter region were not observed between the patients and controls. In conclusion, we identified the core promoter region of *DHX15* and demonstrated that ETS1 and SP1 regulated *DHX15* expression in ALL.

## 1 INTRODUCTION

Acute lymphoblastic leukaemia (ALL) is a heterogeneous haematological malignancy of the bone marrow in which lymphoblasts are overproduced and accumulate.^1^ The aetiology of ALL is unclear; however, aberrant gene expression due to chromosomal translocations, epigenetic abnormalities, activating mutations, hyperdiploidy and altered transcription factors^2^ significantly contributes to this disease. Aberrant expression of *SALL4,* 3 *CXCR4,* 4 *HOX11,* 5 *TAL1,* 5 *LYL1,* 5 *LMO1* 5 and *LMO2* 5 has been implicated in the pathophysiology of ALL. Currently, the primary treatment options for ALL are chemotherapy, radiotherapy and immunotherapy. Patient outcomes have markedly improved; however, the prognosis of ALL is worse in adults than in children. Approximately 50% of adult patients with ALL relapse after the initial treatment. Progress in understanding the pathophysiology of ALL will help advance treatment. Thus, identifying the novel potential targets responsible for ALL pathogenesis may lead to the discovery of novel therapies.

Human RNA helicases (HRHs) are a large family of enzymes that participate in RNA processing. Mutations, aberrant expression and chromosome abnormalities in RNA helicases have been identified in haematological malignancies. *DDX3X* mutations have been identified in chronic lymphocytic leukaemia^6^ and Burkitt's lymphomas,^7^, ^8^ although the exact significance of these mutations is not clear. The *DDX10* gene fuses with the *NUP98* gene to form the chimeric gene *NUP98‐DDX10,* 9 which is involved in de novo or secondary myeloid malignancies^10^, ^11^ as well as imatinib resistance.^12^ The multiplication and self‐renewal of primary human CD34+ cells can also be highly accelerated by NUP98‐DDX10.^13^ Dysregulation of *DDX32* expression has been demonstrated in lymphoid neoplasms,^14^, ^15^ suggesting that this gene may contribute to carcinogenesis. These limited studies reveal that some members of the human RNA helicase family may play diverse biological roles in haematologic malignancies.

The *DHX15* gene (alias *PRP43*) is a member of the DEAH‐box family and is located on the minus strand of chromosome 4 (4p15.3).^16^ Recent evidence has suggested that DHX15 may contribute to carcinogenesis, and overexpression of *DHX15* has been observed in lung adenocarcinoma samples.^17^, ^18^ Semiquantitative RT‐PCR analysis showed up‐regulation of *DHX15* in breast cancer cells. RNAi‐mediated *DHX15* suppression inhibited the proliferation of MCF‐7 and T47D human breast cancer cells.^19^ The DHX15 p.R222G mutation has been identified in de novo or relapsed acute myeloid leukaemia (AML) and myelodysplastic syndrome (MDS) patients and has been implicated as a potential new AML driver gene.^20^, ^21^, ^22^, ^23^ Using real‐time qRT‐PCR and Western blotting, we observed higher *DHX15* mRNA and protein levels, respectively, in human ALL samples than in normal bone marrow (BM) cells. Knockdown of *DHX15* in Jurkat cells leads to impaired cell proliferation and increased apoptosis.^24^ The TSS of the DHX15 gene was identified in a previous study.^16^ However, the transcriptional regulatory mechanism of *DHX15* in ALL remains unknown. Our present study explored the putative promoter region of the *DHX15* gene to characterize the transcriptional regulatory mechanisms of *DHX15* expression in ALL. The results revealed a 501‐bp functional promoter region of *DHX15* that harboured binding sites for ETS1 and SP1, which regulate *DHX15* expression.

## 2 MATERIALS AND METHODS

### 2.1 Cell lines and patient samples

The human T cell leukaemia Jurkat and BCP‐ALL NALM6 cell lines were grown in RPMI 1640 medium containing 10% (v/v) foetal bovine serum (FBS) (Haoyang, Tianjin, China) in a humidified 5% carbon dioxide incubator. Peripheral blood was collected from 121 newly diagnosed patients with ALL (78 males and 43 females; median age 32 [15‐81] years) at Fujian Medical University Union Hospital and from 6 healthy donors (2 males and 4 females; median age 30 [20‐45] years) following the ethical guidelines of our institution and in accordance with the Declaration of Helsinki. All patient samples were obtained prior to the initiation of any therapy. Informed consent was obtained for the procurement and analysis of these specimens.

### 2.2 Plasmid constructs and luciferase reporter assay

Fragments of the *DHX15* promoter truncated at the 5′ end with common sequences at the 3′ end were amplified using PCR. These PCR products were cloned into the XhoI and HindIII sites in a luciferase reporter vector (pGL4.10 [luc]2) (Promega, Madison, WI, USA) lacking a promoter. Positive clones were confirmed by Sanger sequencing. Overlap extension PCR (OE‐PCR)^25^ was used to mutate the ETS1‐ and SP1‐binding sites. The pGL4.10‐345 plasmid was utilized as a template for the first round of OE‐PCR. The presence of the expected mutations in the plasmids was confirmed by Sanger sequencing. All fragments were amplified using the primers listed in Table S1. The Jurkat and NALM6 cells were transiently transfected using the Amaxa Cell Line Nucleofector Kit V (Lonza). A total of 2 × 10^6^ cells were resuspended in 100 μL of Cell Line Nucleofector Solution V and mixed with 4 μg of the promoter constructs and 400 ng of the internal control (pRL‐TK). Nucleofection was performed as previously described.^26^ At 24 hour post‐transfection, the cells were lysed using passive lysis buffer and analysed for luciferase activity with the reagents provided in the Dual‐Luciferase Reporter Assay System (Promega) according to the manufacturer's instructions. The SpectraMax i3x Multi‐Mode detection platform (Molecular Devices, CA, USA) was used to measure the luciferase activity. Firefly luciferase activity was normalized to Renilla luciferase activity and shown as relative luciferase units to reflect the promoter activity.

### 2.3 ETS1 and SP1 constructs

The full‐length cDNA sequence for human *SP1* was obtained by PCR using primers (Table S1) with NheI and HindIII sites. Then, the product was cloned into the pcDNA3.1(‐) vector (Invitrogen, Carlsbad, CA, USA) digested with the same restriction enzymes. Positive clones were validated by sequencing. The pEnter/ETS1 plasmid overexpressing human ETS1 was purchased from VigeneBio (Shandong, China). The expression or empty vectors were transfected into Jurkat and NALM6 cells using the Amaxa Nucleofector.

### 2.4 *In silico* analysis of the *DHX15* gene core promoter

*In silico* analysis of the *DHX15* promoter region between positions −345 and +156 upstream of the transcriptional start site (TSS) was performed with Matinspector (http://www.genomatix.de), Promo Alggen (http://alggen.lsi.upc.es/), TFbind (http://tfbind.hgc.gp/) and MethPrimer (http://urogene.org).

### 2.5 Electrophoretic mobility shift assay (EMSA)

The EMSA was conducted using a chemiluminescence EMSA kit according to the manufacturer's instructions (Beyotime, Jiangsu, China). Briefly, nuclear extracts were prepared from Jurkat and NALM6 cells using the Nuclear and Cytoplasmic Protein Extraction Kit following the manufacturer's instructions (Beyotime). The 5′‐labelled biotin probes corresponding to the putative ETS1 and SP1 sites were synthesized and annealed (Beyotime, Table S1). For regular EMSA, 20 μg of nuclear extract was incubated with the biotinylated probes at 25°C for 30 minutes. To confirm the binding specificity, a 100‐fold excess of unlabelled competitive probe (either a cold probe or a mutated cold probe) was used. To further determine the binding specificity, 4 μg of an antibody that recognized ETS1 or SP1 was also added to the reaction mixture and incubated at 25°C for 30 minutes according to the supershift assay protocol.

### 2.6 Chromatin immunoprecipitation assay (ChIP)

The ChIP assay was performed with the EZ‐Magna CHIP^™^ A kit (Millipore, MA, USA) according to the manufacturer's instructions. Briefly, two million Jurkat and NALM6 cells were harvested, crosslinked, lysed and sonicated (7‐seconds bursts with a 50‐seconds rest period at 20% power using the VibraCell^™^ sonicator). A 1% aliquot of the sample was used as the input DNA control. The remaining cell lysates were incubated with an RNA POL II (Millipore), ETS1 (CST), SP1 (CST) or IgG antibody overnight with rotation. Then, the samples were precipitated using protein A magnetic beads (Millipore). The chromatin‐protein complexes were washed and eluted, and the cross‐linking was subsequently reversed. After purification of the precipitated DNA, the samples were analysed using PCR. The PCR amplifications were performed with 30 cycles at 95°C for 30 seconds, 60°C for 30 seconds and 72°C for 30 seconds. An unrelated region of *GAPDH* was amplified to determine the binding specificity. The DNA primers used for the PCR are listed in Table S1.

### 2.7 RNA interference

Hollenhorst et al^27^ previously described the small interfering RNA (siRNA) target for *ETS1*, and Lars Andresen et al^28^ described the siRNA target for *SP1*. All siRNA duplexes, including a negative control without homology to known human genes, were chemically synthesized by the GenePharma Company (Shanghai, China). The RNAi knockdown experiments were performed with Jurkat and NALM6 cells transiently transfected with the siRNAs using the DharmaFECT 4 transfection reagent (Dharmacon, Inc.) following the manufacturer's instructions. Total RNA and proteins were extracted at 48 hour post‐transfection and analysed using quantitative real‐time PCR (qPCR) and Western blotting, respectively.

### 2.8 Quantitative PCR

Total RNA was extracted using TRIzol (Invitrogen). The concentration and purity of the RNA were determined using a spectrophotometer (NanoDrop 1000, Thermo Scientific). A total of 2 μg of RNA was reverse‐transcribed into cDNA using the PrimeScript RT Reagent Kit (Takara, Dalian, China) and the ABI2720 thermocycler (Applied Biosystems, USA). qPCR was performed with the ABI7500 real‐time PCR system (Applied Biosystems) and FastStart Universal SYBR Green Master Mix (Roche). The primers used for quantification are listed in Table S1. The mean triplicate cT values of each cDNA sample were calculated and then normalized to *GAPDH*. The relative gene expression levels were obtained using the 2^−ΔΔCt^ method as previously described.^29^

### 2.9 Western blotting

RIPA buffer supplemented with the ProteinSafe Protease Inhibitor Cocktail (100×) (TransGenBiotek, China) was used to isolate total proteins from the Jurkat and NALM6 cells. The concentrations of the protein extracts were measured using the BCA Protein Assay Kit (Pierce, Rockford, IL, USA). The Western blotting analysis was performed in the following manner. A total of 20 μg of protein was separated with a 10% sodium dodecyl sulphate (SDS) polyacrylamide gel and then blotted onto PVDF membranes. The membranes were blocked by incubation with 5% skim milk in TBST buffer (10 mmol/L Tris‐HCl, pH 8.0, 150 mmol/L NaCl and 0.1% Tween‐20). After incubation with the anti‐ETS1 (14069, 1:1000 dilution, CST), anti‐SP1 (9389, 1:1000 dilution, CST), anti‐DHX15 (1:1500 dilution, Proteintech) or anti‐β‐actin (1:2000 dilution, TransGen) antibody, the detection was performed with HRP‐coupled secondary antibodies (Millipore). The intensity of each band was calculated using the Quantity One software (Bio‐Rad, Hercules, CA).

### 2.10 BSP

The EpiTect Fast Bisulfite Kit (QIAGEN) was used for bisulphite modification of the genomic DNA. The core promoter region of the *DHX15* gene, which spans a 260‐nucleotide (nt) fragment with 30 CpG sites from −154 to +96 nt, was amplified through two rounds of PCR using primers (Table S1) designed with the Methyl Primer Express Software v1.0 (Applied Biosystems, Foster City, California). Briefly, the first‐round PCR reaction mixture (12.5 μL) contained 1 μL of modified genomic DNA and 6.25 μL of 2× DreamTaq Green PCR Master Mix (Fermentas, MD, USA). The PCR conditions were as follows: 95°C for 5 minutes, followed by 40 cycles of 95°C for 30 seconds, 50°C for 30 seconds and 72°C for 30 seconds with a final elongation at 72°C for 7 minutes. The second‐round amplification reaction (50 μL) contained 2 μL of the diluted first‐round products (1:100) and was conducted under the same conditions. After purification (TIANGEN PCR Purification Kit, Beijing, China), the purified PCR products were cloned into the pGEM‐T vector (Promega, Madison, WI, USA) according to the manufacturer's instructions. Ten independent clones from each specimen were sequenced (SanGon Biotech Co., Shanghai, China). The methylation level for each CpG site was calculated as the number of methylated CpGs in each site divided by the total number of clones sequenced.

### 2.11 Statistical analysis

The patient samples were stratified into low and high DHX15 expression groups based on the median value. The statistical analyses were performed with analysis of variance (ANOVA) and the *t* test in GraphPad Prism 6.02 (GraphPad Software, La Jolla, CA, USA). The Spearman's rho test was used to evaluate the correlation between DHX15 and ETS1 or SP1 expression in the clinical samples. The chi‐square test was used to evaluate the correlations between the clinical characteristics and the DHX15 expression level. *P* values < .05 (two‐sided) were considered significant.

## 3 RESULTS

### 3.1 The *DHX15* core promoter is located within 501‐bp upstream of the transcription start site

**Figure 1:**
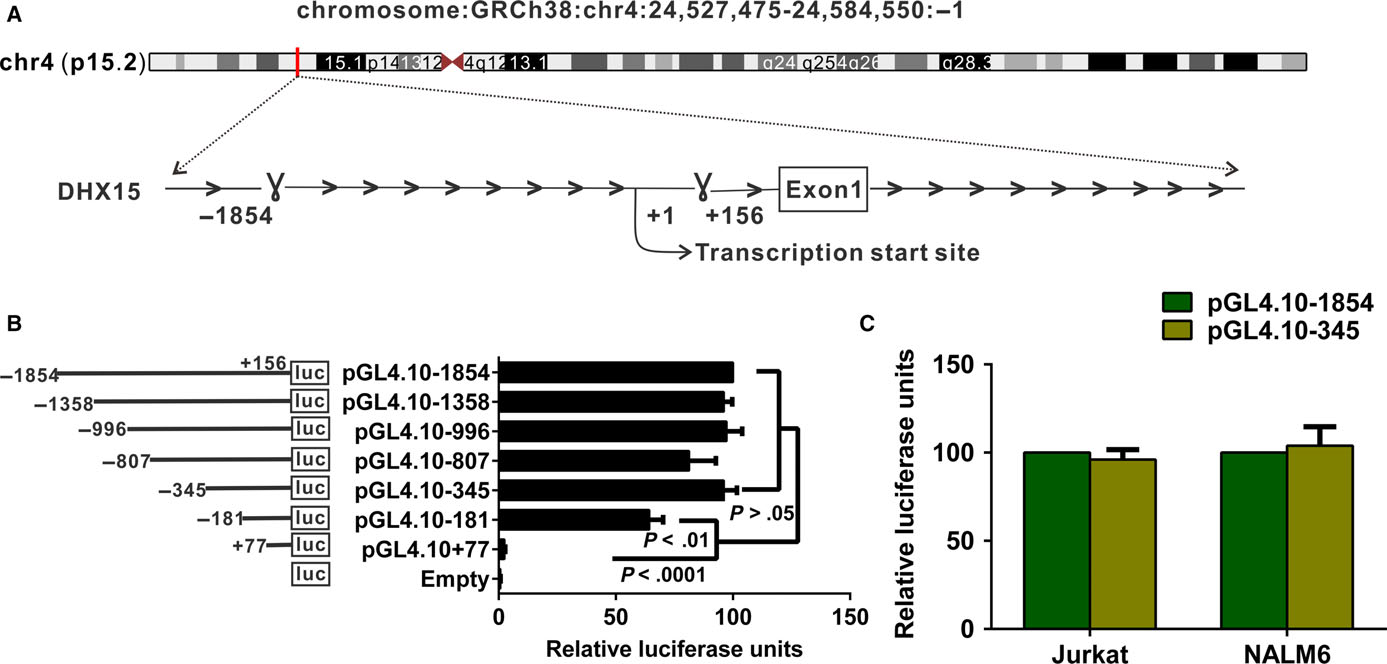
Identification of the DHX15 promoter. A, Schematic representation of the chromosome location and promoter region of the *DHX15* gene. Luciferase assays with the *DHX15* promoter constructs in Jurkat B and NALM6 C, cells. The results represent relative firefly/Renilla luciferase activities, with the activity of pGL4.10‐1854 considered 100%. Values shown are the means ± SDs of three independent experiments

To identify the *DHX15* core promoter, we analysed the 2010‐bp region (−1854 to +156 bp) around the TSS (+1) (Figure 1A). Luciferase experiments suggested that transfection of the two constructs (pGL4.10‐1854 and pGL4.10‐345) into Jurkat cells resulted in stronger luciferase activity than transfection with pGL4.10 alone (both *P* < .0001) (Figure 1B). The difference in luciferase activity between these two constructs was not significant (*P* > .05) (Figure 1B). Additionally, we transfected the pGL4.10‐1854 and pGL4.10‐345 constructs into Jurkat and NALM6 cells. No significant difference in promoter activity was observed between the two constructs in either cell line (Figure 1C). The results suggested that the *DHX15* promoter region spanning from −345 to +156 bp contained the full‐length promoter and was crucial for control of basal *DHX15* expression. Thus, we selected the 501‐bp region for further study.

### 3.2 CpG islands and functional ETS1‐ and SP1‐binding sites are present in the *DHX15* promoter

**Figure 2:**
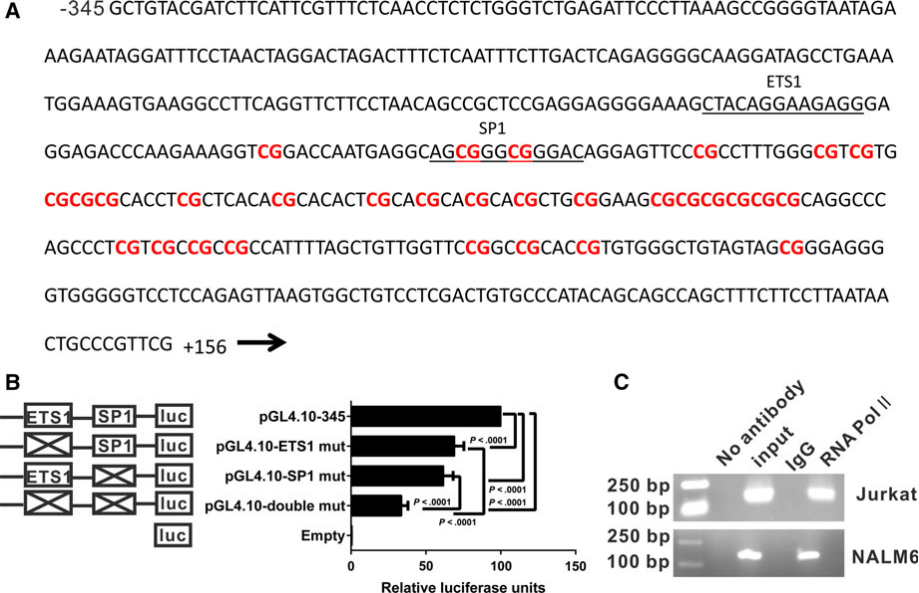
A, The genomic sequence of the *DHX15* core promoter is shown. The sequence spans nucleotides −345 to +156 upstream of the *DHX15* gene. The *DHX15* promoter harbours binding sites for several transcription factors, which are shown underlined, based on predictions from *in silico* programs. Thirty CpG sites (indicated in red) are present in this region. B, Luciferase assays using constructs with mutations in the 501‐bp region with the predicted transcription binding sites in Jurkat cells. The results represent relative firefly/Renilla luciferase activities, with the activity of the WT 501‐bp region considered 100%. The values are expressed as the means ± SDs from three independent experiments. C, Jurkat and NALM6 cell chromatin was immunoprecipitated with an RNA Pol II antibody. Reactions with non‐immune IgG, no antibody and input DNA served as the negative and positive controls. After removal of the cross‐links, the immunoprecipitated DNA was PCR‐amplified using a primer flanking the basal *DHX15* promoter region from −303 to −159 bp. The PCR products were subjected to agarose gel electrophoresis

Comparative analysis using three different software programs (Matinspector, Promo Alggen and TFbind) revealed putative binding sites for several transcription factors in the *DHX15* core promoter region (Figure 2A). We placed emphasis on the ETS1 and SP1 transcription factor binding sites for the following reasons. The ETS1 and SP1 transcription factors drive genes that contribute to proliferation and differentiation. SP1 interacts with TATA‐binding protein‐associated factors (TAFs), which are essential for transcription.^30^ Additionally, SP1 has been implicated in epigenetic regulation^31^ and chemosensitivity^32^, ^33^ in ALL. An ETS1‐binding site was also revealed. ETS1 has been implicated in the pathogenesis of ALL.^34^ Site‐directed mutagenesis suggested that the ETS1 and SP1 transcription factors were essential for *DHX15* promoter activity. A mutation that altered two bases of the ETS1‐binding site reduced the *DHX15* promoter activity to 69% (*P* < .0001) (Figure 2B). Simultaneous mutations of two sites affecting the SP1‐binding site reduced the promoter activity to 62% (*P* < .0001) (Figure 2B). In addition, mutations in the binding sites for both ETS1 and SP1 (designated the pGL4.10‐double mutant) reduced the promoter activity to 34% (Figure 2B), indicating that these transcription factors had an additive effect. The ChIP assay revealed that RNA polymerase II bound to the *DHX15* promoter (−303 to −165) (Figure 2C). CpG islands often contain potential core promoter elements,^35^, ^36^, ^37^ and CpG islands were identified in the *DHX15* core promoter using MethPrimer (Figure 2A).^38^ Taken together, the results suggested that the *DHX15* core promoter was located within the 501‐bp region.

### 3.3 ETS1 and SP1 occupy the *DHX15* promoter

**Figure 3:**
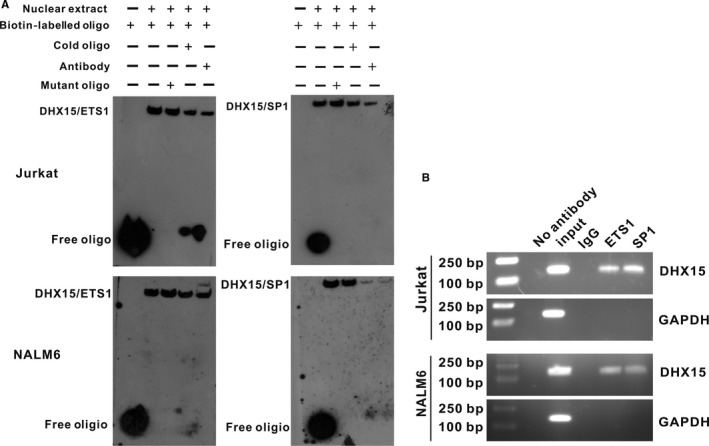
EMSA and ChIP analyses of ETS1 and SP1 binding to the *DHX15* promoter. A, EMSA of ETS1 (left) and SP1 (right). The 5′‐biotin end‐labelled probe was incubated in the absence (lane 0) or presence (lane 1) of nuclear extracts from Jurkat and NALM6 cells. A cold mutated probe (lane 2) and cold probe (lane 3) were used as competitors at concentrations that were in a 100‐fold molar excess to the biotin‐labelled probe. Supershift assays were performed with 4 μg of a specific antibody against ETS1 or SP1 (lane 4). B, Equal amounts of Jurkat and NALM6 chromatin were immunoprecipitated with antibodies for ETS1 and SP1 and subsequently quantified through agarose gel electrophoresis using a primer set specific for the basal region (−181 to −36 bp). Moreover, immunoprecipitated DNA was amplified using a primer set specific to the off‐target region (GAPDH) shown in the lower panel as a negative control

EMSA experiments were performed with nuclear extracts from the Jurkat and NALM6 cells. The complexity of ETS1 (Figure 3A, left) or SP1 (Figure 3A, right) binding to the *DHX15* promoter was demonstrated. Complex formation between the *DHX15* promoter and the ETS1 or SP1 transcription factor was reduced when unlabelled probes were added. The complexity was greatly reduced with the addition of antibodies targeting ETS1 and SP1. In addition, the complexity was unaffected in the presence of mutant probes with two base pair mutations in the binding domain. These results confirmed the binding specificity of these transcription factors for the *DHX15* promoter.

To analyse recruitment of ETS1 and SP1 to the *DHX15* core promoter, we performed a ChIP assay followed by PCR. A clear band was observed in the Jurkat and NALM6 cells, whereas no band was detected in the negative control (Figure 3B). The results indicated *in vivo* occupancy of the *DHX15* core promoter by ETS1 and SP1. The ChIP and EMSA analysis results support the hypothesis that ETS1 and SP1 bind directly to the *DHX15* promoter. Taken together, these results indicate that the DHX15 promoter is a eukaryotic promoter harbouring classical promoter elements, such as CpG islands and ETS1‐ and SP1‐binding sites, which together contribute to transcriptional activation of the *DHX15* gene.

### 3.4 ETS1 and SP1 affect the DHX15 mRNA and protein levels

**Figure 4:**
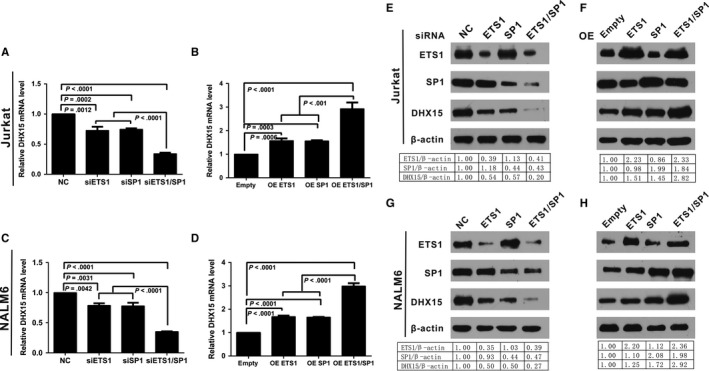
Influence of ETS1 and SP1 on DHX15 gene transcription and protein expression in Jurkat and NALM 6 cells. A, C, E, G, Knockdown of endogenous ETS1 or SP1 or ETS1 and SP1 together decreased DHX15 gene transcription and protein expression. Jurkat and NALM6 cells were transfected with 100 pmol of siRNAs targeting ETS1, SP1 or ETS1 and SP1 together or a negative control (NC). The cells were harvested 48 h after transfection, and 2 μg of the total RNA was used to detect the *DHX15* mRNA level through qPCR. The relative mRNA level was obtained after comparison with the NC, which was set to 1 A, C. Western blotting analysis of total proteins with anti‐ETS1, anti‐SP1 and anti‐DHX15 antibodies was performed for the Jurkat and NALM6 cells; anti‐β‐actin served as a loading control E, G. B, D, F, H, Overexpression (OE) of ETS1 or SP1 or ETS1 and SP1 together increased DHX15 gene transcription and protein expression. Jurkat cells were transfected with 4 μg of pcDNA3.1(‐)/SP1, pEnter‐ETS1, pcDNA3.1(‐)/SP1 and pEnter‐ETS1 together or the empty control pcDNA3.1(‐) pEnter. The cells were harvested 48 h after transfection, and 2 μg of total RNA was used to detect the *DHX15* mRNA level through qPCR. The relative mRNA level was obtained after comparison with the empty vector, which was set to 1 B, D. Western blotting analysis of total proteins with anti‐ETS1, anti‐SP1 and anti‐DHX15; anti‐β‐actin served as a loading control F, H

ETS1 and SP1 were either knocked down or overexpressed to investigate the influence of these transcription factors on the DHX15 gene transcription and protein levels in the Jurkat and NALM6 cells. siRNA‐mediated knockdown of ETS1 or SP1 reduced the DHX15 mRNA (Figure 4A, C) and, consequently, the protein levels (Figure 4E, G). The DHX15 mRNA and protein levels were further reduced by simultaneous knockdown of ETS1 and SP1. In contrast, overexpression of ETS1 or SP1 increased the *DHX15* mRNA levels (Figure 4B, D), and consequently, the DHX15 protein levels (Figure 4F, H) in the Jurkat and NALM6 cells. Overexpression of ETS1 and SP1 further enhanced the DHX15 mRNA and protein levels. Taken together, the current data suggested that ETS1 and SP1 transcriptionally drove *DHX15* expression through their occupancy on the *DHX15* promoter.

### 3.5 The ETS1 and SP1 levels were correlated with *DHX15* expression in ALL

**Figure 5:**
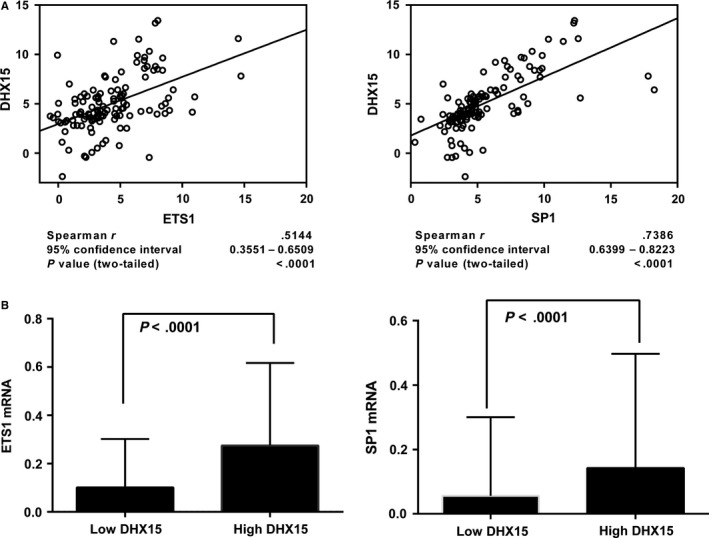
Correlation of the *DHX15* levels with the *ETS1* and *SP1* levels in 121 ALL peripheral blood mononuclear cell (PBMC) samples. A, The qPCR results were evaluated for correlations using Spearman's correlation coefficient, and the correlation coefficient “*r*” was calculated. B, Box plot analyses comparing the *ETS1* and *SP1* levels between samples with low and high *DHX15* expression levels. All qPCR results were normalized to *GAPDH*. The samples were divided into low and high *DHX15* expression groups based on the median value

To assess the correlation between ETS1 and SP1 and *DHX15* in a clinical setting, *DHX15*,* ETS1* and *SP1* expression was investigated using qPCR. Utilizing Spearman's rho, we observed a positive correlation between *DHX15* and either *ETS1* (*r* = .5144) or *SP1* (*r* = .7388), indicating that these two variables were statistically dependent on one another (Figure 5A). We also investigated the biological relevance of *ETS1* and *SP1* for *DHX15*. Patients were stratified into groups with low or high *DHX15* expression based on the median cut‐off value. Remarkably, we observed significant positive correlations of the transcription factor levels with *DHX15*; specifically, patients expressing low *DHX15* levels also showed low expression levels of either *ETS1* or *SP1*, whereas patients with high *DHX15* expression demonstrated high levels of *ETS1* and *SP1* (both *P* < .0001) (Figure 5B). These data further indicated that ETS1 and SP1 regulated *DHX15* expression in human ALL. Additionally, correlations of clinical characteristics and the *DHX15* levels were assessed in the patients with ALL. The *DHX15* expression levels were correlated with peripheral blood blasts (Table S2).

### 3.6 CpG methylation at the *DHX15* promoter is not involved in *DHX15* overexpression in ALL

The methylation status based on bisulphate sequencing of the core promoter region of *DHX15* is shown as lollipop diagrams in Figure S1. Differences in *DHX15* core promoter methylation were not observed between the 6 patients with ALL and the 6 healthy controls (*P* > .05). The CpG sites in the *DHX15* gene core promoter of all samples showed a hypomethylated status.

## 4 DISCUSSION

Recent studies have suggested that *DHX15* mutations can drive leukaemia. Higher DHX15 expression predicts a significantly worse prognosis in multiple myeloma,^39^ breast cancer,^39^ lung adenocarcinoma^39^ and AML.^24^ Transcriptional regulation is crucial for gene expression. However, the mechanism underlying the transcriptional regulation of *DHX15* in ALL is unknown. Our present study provided the first characterization of a 501‐bp core promoter region in the human *DHX15* gene and showed that ETS1 and SP1 directly activated *DHX15* expression.

Luciferase assays of the 2010‐bp region upstream of the TSS of the human *DHX15* gene identified the machinery necessary for activation of basal *DHX15* expression in a 501‐bp region that exhibited full‐length construct activity. Core promoters are often enriched in CpG islands.^35^ The results of our *in silico* analysis showed the presence of CpG islands and ETS1‐ and SP1‐binding sites in this region. The core promoter is the site of action of the RNA polymerase II transcriptional machinery.^35^ Our ChIP assay showed that RNA polymerase II bound to the *DHX15* promoter (−345 to +156). These findings strongly suggested that the core promoter region of *DHX15* was located within the 501‐bp region.

The ETS1 oncogene belongs to a large family within the ETS domain family of transcription factors. ETS1 is involved in the development, differentiation and apoptosis of lymphoid cells. Elevated ETS1 expression has been demonstrated in immature T leukaemic cells^40^ and small lymphocytic cell lymphoma.^41^ Shachar Raz et al^42^ revealed that ETS1 was associated with antifolate resistance. ETS1 has also been implicated in T cell maturation arrest in T‐ALL.^34^ IKKα, which is a subunit of the IkB kinase (IKK) complex, activates nuclear factor‐kB (NF‐kB); ETS1 transactivates IKKα in EU‐4,^43^ which is a B‐cell precursor ALL cell line. SP1 protein expression mediates leukaemogenesis. Significantly elevated SP1 protein levels have been demonstrated in both B‐ALL and T‐ALL samples.^44^ A mouse model of human T‐ALL has been established by overexpressing Notch1 in bone marrow cells.^45^ SP1 drives Notch1 expression in T‐ALL; this finding was confirmed by the observation that SP1 down‐regulation resulted in transcriptional repression of Notch1 in T‐ALL cells.^33^ High hTERT expression has been observed in ALL,^46^ and the SP1 transcription factor plays a critical role in basal hTERT expression in Jurkat T cells^47^; additionally, SP1 regulates hTERT transcription in the leukaemia‐initiating cells of B‐ALL.^48^ These studies show that SP1 is an important mediator that exerts its effects in ALL via downstream signalling molecules.

These findings prompted an investigation of the functional relevance of the binding sites for these transcription factors on the *DHX15* promoter. Site‐directed mutagenesis studies showed that ETS1 and SP1 were putative transcriptional regulators of the *DHX15* promoter. The EMSA analysis results confirmed the *in vitro* interaction of these transcription factors with the *DHX15* promoter. Using the ChIP assay, we confirmed that these interactions occurred *in vivo*. Overexpression and RNAi studies confirmed that the DHX15 gene was transcriptionally regulated by ETS1 and SP1. Dysregulation of DHX15 as an interacting partner with other proteins has been studied in breast cancer^19^ and prostate cancer.^49^ Here, we contribute to the understanding of the protein‐DNA interactions of the ETS1 and SP1 transcription factors with the *DHX15* gene promoter.

We assessed the association of ETS1 and SP1 with *DHX15* in clinical samples. A positive correlation between *DHX15* and *ETS1* and between *DHX15* and *SP1* was observed in the patients with ALL. Patients with high *DHX15* expression also had high *ETS1* and *SP1* expression levels. These results further support the notion that ETS1 and SP1 regulate DHX15 expression. Additionally, the correlation between peripheral blood blasts and the *DHX15* expression level suggests that the *DHX15* expression level possibly predicts the leukaemia burden in the peripheral blood. BSP showed that the *DHX15* core promoter was hypomethylated in the patients with ALL and in healthy controls. SP1 maintains the hypomethylated status of CpG islands.^36^ Therefore, we postulated that the presence of a SP1‐binding site in the *DHX15* core promoter contributed to maintenance of the hypomethylated status of the *DHX15* core promoter and active *DHX15* expression.

Our present study is the first to report that the human RNA helicase DHX15 may be transcriptionally regulated in ALL by the ubiquitous transcription factors ETS1 and SP1. The two transcription factors are particularly promising therapeutic targets because these proteins mediate signals from multiple pathways. Targeting these transcription factors will likely interfere with critical signalling molecules in haematologic malignancies. The present study enhances our current understanding of the transcriptional regulation of *DHX15* in ALL; however, additional studies are needed to uncover the complex transcriptional mechanisms underlying *DHX15* expression.

## CONFLICT OF INTEREST STATEMENT

The authors confirm that there are no conflicts of interest to disclose.

## AUTHOR CONTRIBUTIONS

XLC, YHC, QL, LLP, SLS and JW performed the cellular and molecular experiments. XLL, JGL and YC collected the blood samples. XLC, YHC, YL and XFL performed the data analysis. XLC, YHC and QL wrote the manuscript. SYW designed and supervised this study. SYW obtained financial support.
