# Supplementary material for: ETS1 and SP1 drive DHX15 expression in acute lymphoblastic leukaemia
Source: J Cell Mol Med. 2018 Mar 7;22(5):2612–21. doi: 10.1111/jcmm.13525 (PMC5908128; doi:10.1111/jcmm.13525)
Supplement: Supplementary file 1 [file JCMM-22-2612-s001.docx]

| **Table 1.** List of oligonucleotides used in the present study^a^. | | | |
| --- | --- | --- | --- |
| **Promoter cloning**  sequence (5’ 3’) Length of products | | | |
| -1854/+156F | ATG *CTCGAG* GCCATCACCCCATCATAAGTCA | | 2010 bp |
| -1358/+156F | ATCG *CTCGA* GGCTGAGATTGCACCACTGTCCT | | 1514 bp |
| -996/+156F | ATCG *CTCGAG* TGTAACCTTCCTCAGCCTTTGC | | 1152 bp |
| -807/+156F | ATCG CTCGAG CTGGGCAAGAAAAGGGCAAA | | 963 bp |
| -345/+156F | ATCG CTCGAG GCTGTACGATCTTCATTCGTTTC | | 501 bp |
| -181/+156F | ATCG CTCGAG CTTCCTAACAGCCGCTCCGA | | 337 bp |
| +77/+156F | ATCG CTCGAG GGGTCCTCCAGAGTTAAGTGGC | | 79 bp |
| -1854/+156R | CCC AAGCTT CGAACGGGCAGTTATTAAGGAA | |  |
| **Site-directed mutagenesis** |  | |  |
| ETS1 mut F | GGGAAAGCTACAGACAGAGGGAGGAGACC | |  |
| ETS1 mut R | GTCTCCTCCCTCTGTCTGTAGCTTTCCCC | |  |
| SP1 mut F | CCAATGAGGCAGCGTACGGGACAGGAGTTC | |  |
| SP1 mut R | GAACTCCTGTCCCGTACGCTGCCTCATTGG | |  |
| **Transcription factor cloning** | |  |  |
| SP1 F | CTA *GCTAGC* GCCACC ATGAGCGACCAAGATCAC | |  |
| SP1 R | CCC *AAGCTT* GAAGCCATTGCCACTGATATT | |  |
| **EMSA** |  | |  |
| ETS1 oligo F | 5’ biotinGGGAAAGCTACAGGAAGAGGGAGGAGAC | |  |
| ETS1 oligo R | 5’ biotinGTCTCCTCCCTCTTCCTGTAGCTTTCCCC | |  |
| SP1 oligo F | 5’ biotinCCAATGAGGCAGCGGGCGGGACAGGAGTTC | |  |
| SP1 oligo R | 5’ biotinGAACTCCTGTCCCGCCCGCTGCCTCATTGG | |  |
| ETS1 mut F | GGGAAAGCTACAGACAGAGGGAGGAGAC | |  |
| ETS1 mut R | GTCTCCTCCCTCTGTCTGTAGCTTTCCCC | |  |
| SP1 mut F | CCAATGAGGCAGCGTACGGGACAGGAGTTC | |  |
| SP1 mut R | GAACTCCTGTCCCGTACGCTGCCTCATTGG | |  |
| **CHIP** |  | |  |
| ETS1/ SP1 CHIP F | CTTCCTAACAGCCGCTCCGA | | 147 bp |
| ETS1/ SP1 CHIP R | TGCGAGTGTGCGTGTGAGCGAG | |  |
| RNA POL2 CHIP F | GATTCCCTTAAAGCCGGGGT | | 137 bp |
| RNA POL2 CHIP R | GCGGCTGTTAGGAAGAACCT | |  |
| **Real-time RT-PCR** |  | |  |
| ETS1 F | AGACCCTCTCCAGACAGACA | | 158 bp |
| ETS1 R | GGCGATCACAACTATCGTAGCT | |  |
| SP1 F | AAGAAATGACCTTAGGAACATAC | | 133 bp |
| SP1 R | CCGTATATGTCTACACACAGATG | |  |
| DHX15 F | TCTACACTTCCACCTCAGCAGCA | | 152 bp |
| DHX15 R | CCAGGATCAATCACAAACACCACAC | |  |
| GAPDH F | CCATCACCATCTTCCAGGAGCG | | 149 bp |
| GAPDH R | AGAGATGATGACCCTTTTGGC | |  |
| **Bisulfite sequencing PCR (BSP)** |  | |  |
| DHX15 1st F | TTTTGGGTTTGAGATTTTTTTA | |  |
| DHX15 1st R | ACAACCACTTAACTCTAAAAAACCC | |  |
| DHX15 2nd F | AAAGTTATAGGAAGAGGGAGGAGATT | |  |
| DHX15 2nd R | ACTTAACTCTAAAAAACCCCCA | |  |
| ^a^Mutant bases are underlined. Restriction sites are shown in italics. | | | |

**Table 2.** Comparison of the clinical characteristics and outcomes of adolescent or adult ALL patients with respect to DHX15 expression.

| Characteristics | No. of cases | *DHX15*-low  n(%) | *DHX15*-high  n(%) | P values |
| --- | --- | --- | --- | --- |
| **Median age at diagnosis, years (range)**  32(15-81) | | | | |
| **Age (years)** 0.712 | | | | |
| 15-35 | 71 | 37(52) | 34(48) |  |
| 36-55 | 33 | 17(51.5) | 16(48.5) |  |
| ≥ 56 | 17 | 7(41) | 10(59) |  |
| **Sex**  0.9025 | | | | |
| Male | 78 | 39(50) | 39(50) |  |
| Female | 43 | 22(51) | 21(49) |  |
| **WBC (×10^9^/l)**  0.901 | | | | |
| < 30 | 54 | 28(52) | 26(48) |  |
| 30-100 | 35 | 17(49) | 19(51) |  |
| > 100 | 31 | 16(52) | 15(48) |  |
| **Percentage of PB blast** **0.0121** | | | | |
| < 60% | 50 | 32(64) | 18(36) |  |
| ≥ 60% | 71 | 29(41) | 42(59) |  |
| **ALL subtype**  0.8646 | | | | |
| T | 34 | 17(50) | 17(50) |  |
| B | 87 | 45(52) | 42(48) |  |
| **B-ALL^a^** |  |  |  | 0.2269 |
| ph-chromosome - | 27 | 10(37) | 17(63) |  |
| ph-chromosome+ | 69 | 35(51) | 34(49) |  |
| **Response to induction therapy^b^**  0.5639 | | | | |
| CR | 73 | 36(49) | 37(51) |  |
| Failure | 25 | 14(56) | 11(44) |  |

Abbreviations: WBC=white blood cell count, PB=peripheral blood, CR=complete remission.

^a^ one patient was not subjected to chromosome analysis.

^b^Due to lose of follow up, early death and lack of systemic therapy due to poor economic conditions, therapy-related information could not be obtained for 23 patients.

**
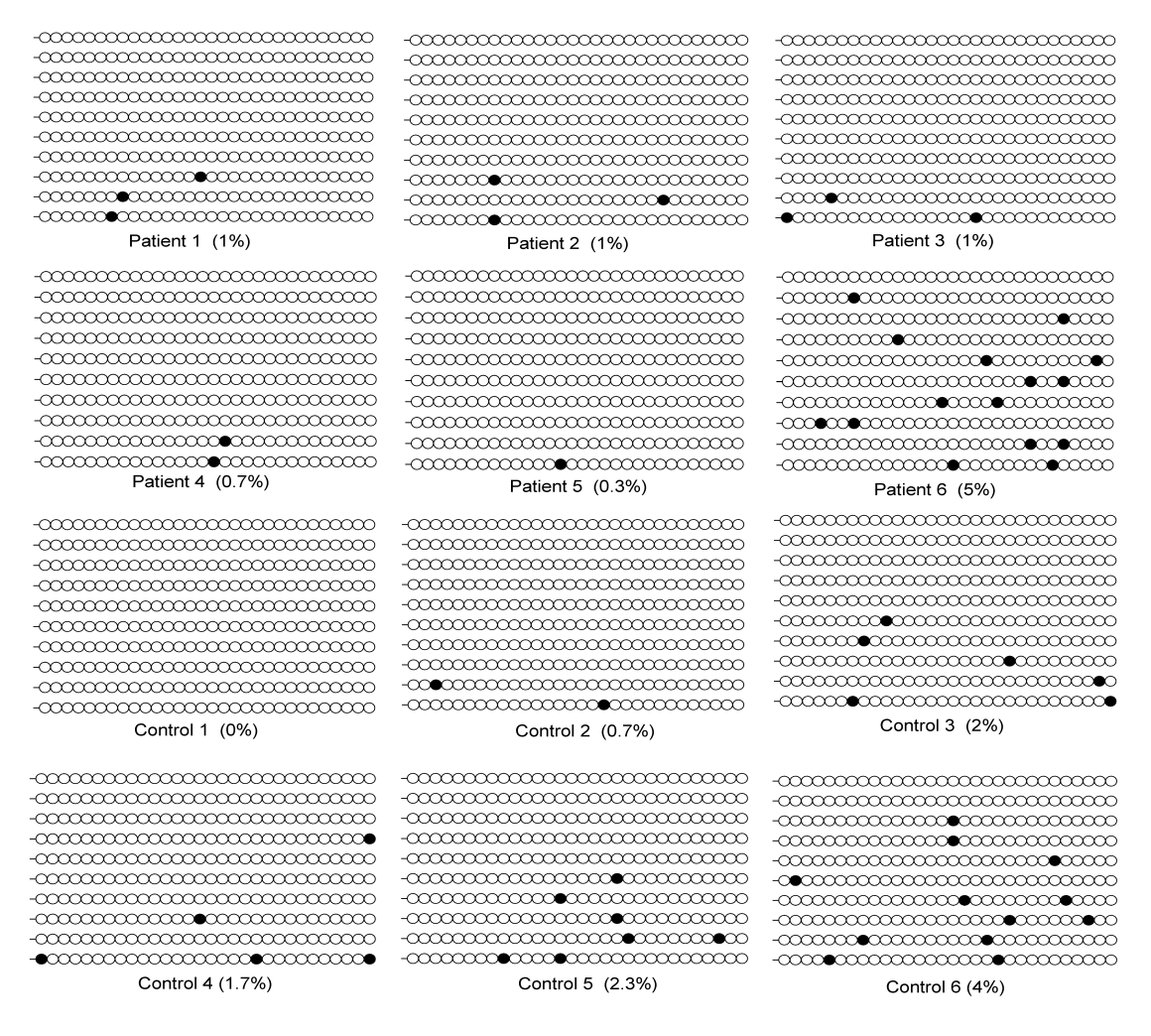
**

**Fig. 1.** DHX15 methylation density in six ALL patients and six healthy controls.

White cycle: unmethylated CpG dinucleotide; Black cycle: methylated CpG dinucleotide.
